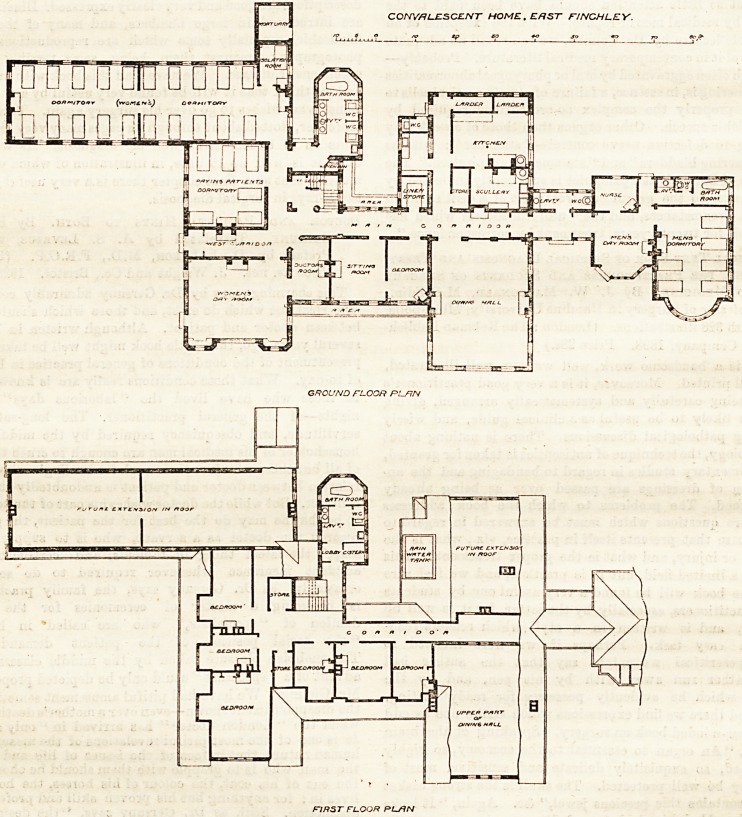# Hospital Construction

**Published:** 1898-05-07

**Authors:** 


### The Institutional Workshop.

### HOSPITAL CONSTRUCTION.

### CONVALESCENT HOME AT EAST FINCHLEY.

A home for convalescents has recently been erected at East Finchley in connection with the National Hospital for the Paralysed and Epileptic, Queen Square, to take the place of the two small villas which have been used for women convalescents for many years. The buildings, which were opened by H.R.H. the Duchess of Albany on January 16th, contain accommodation for six men, for twenty ordinary female patients, and six paying female patients, together with some cots for children. The accompanying plan shows the arrangement of the building. All the rooms which are to be used by the patients are on the ground floor, partly because it is not desirable for those suffering from epilepsy to have to go up and down stairs, partly so as to facilitate the moving of paralysed patients in wheel chairs from room to room. It will be noticed that the dining hall and the kitchen, scullery, larder, and store-room are all grouped together, and stand between the men's side and the women's, being equally accessible from each. The men's department contains a dormitory with six beds, a bath-room, two lavatories and w.c.'s, and a room for a nurse, while on the women's side there are two dormitories and a small isolation-room. Probably the latter is only intended for the temporary separation of a patient during or after a fit. If wanted for use by a patient who requires to be really isolated it should have been much more completely separated than it is from the dormitory. The sanitary arrangements on the women's side are well placed in a separate block. This, however, might have been much more satisfactorily cut off from the rest of the house than it is if a window in the lobby leading to it had not been appropriated for a foul linen cupboard. On the men's side there are two sets of sanitary arrangements, the reason for which we do not quite see, and the position of one of them close to the dormitories is not altogether satisfactory.

The store-room by the side of the scullery seems to be quite dark, nor do we see exactly how the two store-rooms upstairs are to be lighted. With these exceptions the building seems to be carefully planned and very well fitted for its present purpose. In the roof of the central portion of the building there is a series of bedrooms for the staff, with bath-rooms, &c., and in other parts of the roof space is left for future extensions. The elevation is extremely picturesque, and the roof is a strongly marked feature in it. We cannot but think, however, that a few courses more walling would have materially improved the upstairs rooms, and the proposed future extension.

## Figures and Tables

**Figure f1:**